# Balancing immunotherapy and corticosteroids in cancer treatment: dilemma or paradox?

**DOI:** 10.1093/oncolo/oyaf045

**Published:** 2025-03-31

**Authors:** Alessandro Ottaiano, Mariachiara Santorsola, Maurizio Capuozzo, Stefania Scala

**Affiliations:** SSD-Innovative Therapies for Abdominal Metastases, Istituto Nazionale Tumori di Napoli, IRCCS Fondazione “G. Pascale,” 80131 Naples, Italy; SSD-Innovative Therapies for Abdominal Metastases, Istituto Nazionale Tumori di Napoli, IRCCS Fondazione “G. Pascale,” 80131 Naples, Italy; Coordinamento Farmaceutico, ASL-Naples-3, 80056 Ercolano, Italy; Microenvironment Molecular Targets, Istituto Nazionale Tumori di Napoli, IRCCS Fondazione “G. Pascale,” 80131 Naples, Italy

## Abstract

Corticosteroids are widely used to prevent and treat chemotherapy-induced nausea and vomiting (CINV) due to their pleiotropic biological effects. However, concerns have been raised about their immunosuppressive properties when combined with immunotherapy. Specifically, their potential impact on the efficacy of immunotherapy, mainly immune checkpoint inhibitors (ICIs), remains a subject of debate. This manuscript discusses the mechanisms by which corticosteroids mitigate CINV, the challenges associated with their concurrent use with immunotherapy, and emerging therapeutic strategies evaluating dexamethasone-free regimens. A careful balance must be struck in corticosteroid use to effectively manage CINV while optimizing the outcomes of immunotherapy.

## Introduction

Chemotherapy-induced nausea and vomiting (CINV) remains one of the most significant acute and delayed side effects impacting cancer patients’ quality of life. Corticosteroids, particularly dexamethasone, have demonstrated considerable efficacy and were quickly incorporated into CINV treatment protocols. Notably, corticosteroids possess pleiotropic biological effects, contributing to their wide therapeutic utility in CINV. The scientific community has focused not only on identifying increasingly effective drugs and their combinations but also on optimizing their therapeutic efficacy.

## Challenges in combining corticosteroids and immunotherapy

Monoclonal antibodies targeting the immune checkpoints (Immune Checkpoints Inhibitors, ICIs) have become widely used in various malignancies.^1^ The PD-1 (Programmed Cell Death-1)/PD-L1 (Programmed Cell Death-Ligand 1) pathway is a key target immune checkpoint pathway. PD-1 is a receptor expressed on activated T cells, B cells, and other immune cells playing a pivotal role in suppressing their activity. It binds to PD-L1 and PD-L2, which are frequently overexpressed on tumor cells. Many tumors exploit this pathway to escape immune surveillance and destruction. By blocking the PD-1/PD-L1 interaction, antibodies prevent the inhibitory signals that limit T-cell function. Pembrolizumab and nivolumab target PD-1, and durvalumab, targets PD-L1. Similarly, ipilimumab and tremelimumab inhibit CTLA-4 (Cytotoxic T-Lymphocyte-Associated Protein 4), another immune checkpoint. CTLA-4 acts as a negative regulator of T-cell activation by competing with the stimulatory receptor CD28 for binding to CD80 and CD86 on antigen-presenting cells (APCs). Engagement of CTLA-4 reduces early T-cell activation in the lymph nodes, affecting the priming of naïve T cells and promoting immune tolerance. By blocking CTLA-4, these antibodies enhance T-cell activation and proliferation.^2^ However, amidst the optimism surrounding immunotherapeutic strategies, there remain many unanswered questions such as the failure of immunotherapy in many tumor types and the emergence of resistance.^3,4^ The administration of ICIs in conjunction with specific drugs poses a crucial issue. For example, ICIs provoke drug-induced hepatitis when given in combination with pazopanib.^5^ Another combination that has raised concern is the practice of administering immunotherapeutics after dexamethasone or other corticosteroids, which are commonly used as anti-emetic premedications.

## Corticosteroids in CINV management: mechanisms and concerns

Corticosteroids exert anti-emetic effects through multiple mechanisms of action. A key mechanism is the reduction of inflammation inhibiting the release of pro-inflammatory cytokines and neuropeptides involved in emesis, such as neurokinin-1 (NK1).^6^ Additionally, corticosteroids may affect the hypothalamic-pituitary-adrenal (HPA) axis, potentially mitigating adrenal insufficiency caused by chemotherapy and alleviating nausea linked to low cortisol levels.^7^ Furthermore, corticosteroids may counteract the effects of chemotherapeutic agents induced molecules, such as platinum compounds increase GDF-15, which evoke nausea and food aversion.^8^ Lastly, corticosteroids exhibit pleiotropic anti-emetic effects on the gastrointestinal tract, promoting gastric motility and reducing visceral hypersensitivity. These mechanisms highlight the multifaceted role of dexamethasone in managing CINV, reinforcing its importance in anti-emetic regimens.^9,10^ Although the majority of immunotherapy study protocols and recommendations prohibit the use of corticosteroids prior to the administration of ICIs, in practice, this is difficult to pursue mainly in combined regimens with chemotherapy or in the management of brain metastases. This may potentially affect the efficacy of ICIs.^11^

## Evidence of corticosteroids’ impact on immunotherapy efficacy

While dexamethasone is effective in mitigating CINV,^12^ the immunosuppressive properties raise significant concerns about the potential impact on the efficacy of immunotherapy, although direct evidence are not available due to the lack of prospective comparative studies on the effect of steroid use on immunotherapy outcomes.^13^ Notably, in a comprehensive retrospective study involving 640 patients with non-small cell lung cancer (NSCLC) treated with single-agent PD-1/PD-L1 inhibitors (pembrolizumab, nivolumab, atezolizumab, or durvalumab), baseline corticosteroids (oral or intravenous ≥10 mg prednisone equivalent) on the first day of ICIs correlated with worse clinical outcomes. Specifically, corticosteroid administration (≥10 mg vs <10 mg) at the start of PD-1/PD-L1 blockade was associated with a reduced objective response rate (odds ratio, 0.42; *P* = .053) and significantly shortened progression-free survival (HR, 1.31; *P* = .03) and overall survival (HR, 1.66; *P* < .001).^14^ Conversely, a case report on advanced malignant melanoma treated with ipilimumab highlighted that long-term oral corticosteroid therapy with methylprednisolone (80 mg daily), used to manage immune-related adverse events (irAEs) such as diarrhea, fatigue, nausea, and vomiting, did not affect the response to ipilimumab for over 2 years.^15^

**Figure 1.**
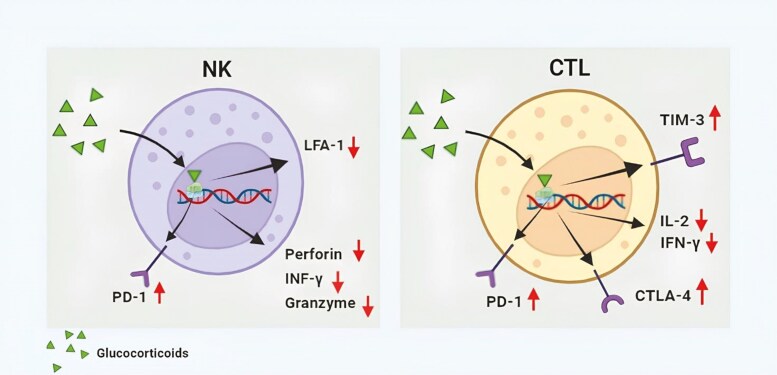
Glucocorticoids are highly lipophilic substances, allowing them to rapidly penetrate (within minutes) across cellular membranes. Once inside the nucleus, they bind to the glucocorticoid receptor (GR), which subsequently modulates the transcription of target genes. This complex can either activate or repress gene transcription, affecting various cellular functions. The figure reports the most well-known effects, with potentially significant implications for the recognition and elimination of tumor cells. In natural killer (NK) cells, transcription of programmed cell death-1 (PD-1) is upregulated, while lymphocyte function-associated antigen-1 (LFA-1), perforin, interferon-gamma (IFN-γ), and granzyme are reduced. In cytotoxic T lymphocytes (CTLs), PD-1, cytotoxic T-lymphocyte-associated protein 4 (CTLA-4), and T-cell immunoglobulin and mucin-domain containing-3 (TIM-3) are upregulated, whereas interleukin-2 (IL-2) and IFN-γ are downregulated. These effects limit the cytotoxic properties of both NKs and CTLs against tumor cells.

A key concern arises from the inherent immunosuppressive nature of dexamethasone and other corticosteroids. Corticosteroids exert potent anti-inflammatory and immunomodulatory effects, suppressing various components of the immune system.^16,17^ Thus, corticosteroid are invaluable in the management of inflammatory, autoimmune conditions, and antirejection regimens in the setting of transplantation,^18^ but a fundamental contradiction appears when administered concomitantly with immunotherapy, which seeks to harness and enhance the immune response against cancer cells. Moreover, a more profound concern arises regarding the potential antagonistic effect of corticosteroids on the anti-tumor activity of both chemotherapy and chemo-immunotherapy combinations. While this concern is particularly pertinent to chemo-immunotherapy, the role of the immune response in various tumors, mainly nonresponsive to PD-1/PD-L1 inhibitors, remains largely unknown. Given their ability to dampen immune activation, cytokines, and PD-1 expression,^19^ corticosteroids may undermine the therapeutic efficacy of any therapeutic agent, thereby compromising treatment outcomes. In phase III randomized clinical trials combining immunotherapy and chemotherapy, premedication for CINV is permitted according to local prescribing information. However, immunosuppressive medications such as systemic corticosteroids are generally prohibited, except in specific cases like documented hypersensitivity reactions. This approach is outlined for chemo-immunotherapy combinations in biliary tract tumors (NCT03875235), lung cancer (NCT0383815), and ovarian cancer (NCT03038100). Thus, the potential negative impact of corticosteroids on the efficacy of immunotherapy is already recognized, even though their use as premedication for CINV is not explicitly prohibited. Figure 1 illustrates the effects of glucocorticoids on key effector cells from both innate immunity (NK cells) and adaptive immunity (T cells) that are primarily involved in recognizing and eliminating tumor cells. This leads to a fundamental question: are corticosteroids truly indispensable and irreplaceable in the prevention of CINV?

## Historical evolution of anti-emetic regimens

**Figure 2.**
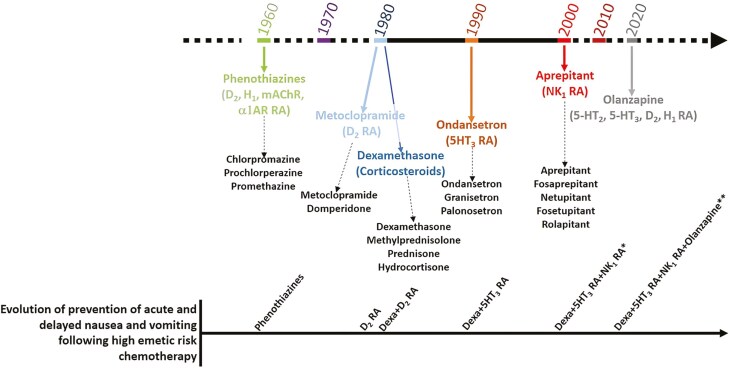
Evolution over time of anti-emetic drugs and their combinations (α1AR, alpha 1 adrenergic receptor; D2, Dopamine D2 receptor; 5HT2, Serotonin 5-HT2 receptor; 5HT3, Serotonin 5-HT3 receptor; H1: Histamine H1 receptor; mAChR: muscarinic Acetylcholine Receptors; NK1, Neurokinin-1 receptor; RA, Receptor Antagonist). *Including NEPA (NK1RA: fosnetupitant, and 5-HT3RA: palonosetron), a regimen extensively studied in the literature. **A four-drug regimen consisting of a single dose of a 5HT3-RA, dexamethasone, an NK1RA (such as aprepitant, fosaprepitant, netupitant, fosnetupitant, or rolapitant), and olanzapine administered before chemotherapy is recommended for the prevention of acute nausea and vomiting following highly emetogenic chemotherapy (MASCC/ESMO guidelines).

The management of CINV has undergone significant evolution, marked by the development and integration of various anti-emetic agents. Phenothiazines were the first class of drugs introduced in the 1960s for use as antiemetics.^20^ By the early 1980s, metoclopramide, which primarily acts by blocking dopamine D2 receptors in the chemoreceptor trigger zone (CTZ) of the brain, emerged as a new and effective antiemetic agent.^21^ During the same period, steroids were studied and shown to be efficacious as antiemetics. Dexamethasone emerged as a cornerstone treatment due to its efficacy both as monotherapy and in combination with other agents in reducing CINV.^22-25^ This agent, despite its broad physiological impact, proved crucial in the premedication regimens to mitigate CINV. In the 1980s, the advent of 5-HT_3_ receptor antagonists such as ondansetron, represented a major advancement. These drugs specifically targeted the serotonin receptors implicated in CINV, offering a targeted approach with fewer side effects compared to corticosteroids. The combination of corticosteroids with 5-HT_3_ antagonists significantly improved patient outcomes, providing a more robust anti-emetic coverage.^26^ The 1990s and early 2000s brought further refinement with the introduction of NK_1_ receptor antagonists including aprepitant and fosaprepitant. These drugs addressed the neurokinin-1 pathway, another critical component in the emetic response. The integration of NK_1_ receptor antagonists into existing regimens of corticosteroids and 5-HT_3_ antagonists resulted in a “triple therapy.”^27^ The effectiveness of NEPA (netupitant–palonosetron)-based regimens to aprepitant-based regimens was evaluated in managing CINV, finding NEPA-based therapies more effective in preventing CINV, especially during the delayed and overall phases following chemotherapy.^28^ Importantly, the totality of the studies included NEPA plus dexamethasone. In their discussion, they underscored the necessity of evaluating the potential adverse effects of dexamethasone on the immune system, particularly in the context of immunotherapy. More recently, the addition of atypical antipsychotics such as olanzapine has offered further improvements. Olanzapine, due to its action on multiple neurotransmitter receptors, enhances the anti-emetic effect when combined with the standard triple regimen, providing better control over both acute and delayed phases of CINV. This “four-drug” regimen is now recommended by ASCO and MASCC/ESMO guidelines for highly emetogenic chemotherapy regimens^29^ (Figure 2).

## Reassessing the role of corticosteroids in CINV management and optimizing treatment approaches

Given the enhanced understanding of the biological pathways involved in emesis mechanisms and the availability of new drugs, dexamethasone-free antiemetic regimens are strongly sought. A double-blind, phase III trial demonstrated that a dexamethasone-free regimen (olanzapine, palonosetron, and fosaprepitant vs olanzapine, palonosetron, and dexamethasone) is not only non-inferior-but-superior in preventing CINV compared to the regimen containing dexamethasone.^30^ In the dexamethasone-free regimen, patients received olanzapine at a dose of 5 mg orally, administered once daily on days 1, 2, 3, and 4, starting 30 minutes before the first chemotherapy administration on day 1. Palonosetron was given intravenously at a dose of 0.25 mg as a bolus 30 minutes before the chemotherapy infusion. Additionally, fosaprepitant was administered intravenously at a dose of 150 mg, infused in 150 mL of 0.9% normal saline, with the infusion completed 30 minutes prior to chemotherapy. This carefully structured regimen optimized the management of nausea and vomiting, providing patients with a comprehensive anti-emetic strategy throughout their treatment. Other randomized studies are evaluating the efficacy of dexamethasone-free regimens against CINV (NCT03040726, NCT03578081).

A recent meta-analysis has shown that omitting dexamethasone from antiemetic regimens does not result in a significant loss of protection against CINV.^31^ In immune active cancers, corticosteroid-induced immunosuppression could affect responses to immunotherapeutic drugs and prognosis (melanoma, non-small cell lung cancer, renal cell carcinoma, and lymphoma).

## Conclusion: addressing the dilemma and paradox

While the advent of immunotherapy represents a transformative milestone in cancer treatment, careful consideration of adjunctive therapies such as dexamethasone is imperative to maximize treatment efficacy. A dilemma arises from the need to balance the benefits of corticosteroids in managing chemotherapy side effects, which may potentially compromise immunotherapy efficacy. Furthermore, a paradox persists in leveraging immunotherapy to enhance the immune response against cancer cells while concurrently administering immunosuppressive corticosteroids, potentially undermining treatment outcomes. A growing number of experts have suggested steroids given as antiemetics may be detrimental.^11,32,33^ An important recommendation that can optimize the interference window between anti-emetic and immunosuppressive effects arises from the recent joint update of the MASCC/ESMO guidelines on the prevention of CINV following high-emetic-risk antineoplastic agents: a single day of dexamethasone is as effective as a 3-day regimen.^34^ This approach can be particularly beneficial in mitigating the immunosuppressive effects of dexamethasone in combination regimens involving immunotherapy and chemotherapy.

In this transitional phase, to alleviate concerns surrounding the concurrent use of corticosteroids and immunotherapy, several strategic interventions merit consideration. First, the necessity and harmonization of corticosteroid dosages as anti-emetic premedication is imperative, given the considerable heterogeneity observed in clinical practice. Healthcare providers should exercise prudence in prescribing corticosteroids, opting for the lowest effective dose and duration to minimize potential immunosuppressive effects while still adequately managing chemotherapy-induced side effects. Furthermore, alternative anti-emetic strategies that circumvent the use of corticosteroids should be explored and implemented where feasible.^35^ Non-pharmacological interventions, including behavioral therapies, music therapy, and dietary management, combined with anti-emetic agents that have minimal immunosuppressive properties, such as 5-HT3 receptor antagonists, neurokinin-1 receptor antagonists, and olanzapine, may offer effective alternatives for managing CINV without compromising the immune response.^36,37^ We suggest that future studies consider measuring serum cortisol levels in patients undergoing cancer treatments to select patients most likely to benefit from corticosteroids.^38,39^ Additionally, we propose that trials need to randomize patients to different doses of steroids. Investigating their potential dose-dependent effects on CINV could yield significant insights and help establish optimal dosing strategies. The scientific community often underestimates the critical evaluation of the potential immunosuppressive effects of corticosteroids and the implementation of strategic measures to mitigate their impact on immunotherapy outcomes. However, we believe these are essential steps toward optimizing patient care and advancing cancer therapeutics.

## Data Availability

No new data were generated or analysed in support of this research.
